# Quantitative ubiquitylomics reveals the ubiquitination regulation landscape in oral adenoid cystic carcinoma

**DOI:** 10.1042/BSR20211532

**Published:** 2021-08-20

**Authors:** Wen Li, Xiaobin Wang, Qian Zhang, Hanlin Wang, Wenxin Zuo, Hongliang Xie, Jianming Tang, Mengmeng Wang, Zhipeng Zeng, Wanxia Cai, Donge Tang, Yong Dai

**Affiliations:** 1 Carson International Cancer Centre, Shenzhen University General Hospital and Shenzhen University Clinical Medical Academy Centre, Shenzhen University, 1098 Xueyuan Road, Shenzhen Guangdong 518000, China; 2 Key Laboratory of Optoelectronic Devices and Systems, College of Physics and Optoelectronic Engineering, Shenzhen University, Shenzhen 518060, China; 3 Health Science Center, School of Medicine, Shenzhen University, Shenzhen 518060, China; 4 Clinical Medical Research Center, Guangdong Provincial Engineering Research Center of Autoimmune Disease Precision Medicine, Shenzhen Engineering Research Center of Autoimmune Disease, The Second Clinical Medical College of Jinan University, The First Affiliated Hospital of Southern University of Science and Technology, Shenzhen People’s Hospital, Shenzhen, Guangdong 518020, China

**Keywords:** Adenoid Cystic Carcinoma, biomarkers, Protein Ubiquitination, proteomics, PTM, therapeutics

## Abstract

Adenoid cystic carcinoma (ACC) is an extremely rare salivary gland tumor with a poor prognosis and needs attention on molecular mechanisms. Protein ubiquitination is an evolutionarily conserved post-translational modification (PTM) for substrates degradation and controls diverse cellular functions. The broad cellular function of ubiquitination network holds great promise to detect potential targets and identify respective receptors. Novel technologies are discovered for in-depth research and characterization of the precise and dynamic regulation of ubiquitylomics in multiple cellular processes during cancer initiation, progression and treatment. In the present study, 4D label-free quantitative techniques of ubiquitination proteomics were used and we identified a total of 4152 ubiquitination sites in 1993 proteins. We also performed a systematic bioinformatics analysis for differential modified proteins and peptides containing quantitative information through the comparation between oral ACC (OACC) tumor with adjacent normal tissues, as well as the identification of eight protein clusters with motif analysis. Our findings offered an important reference of potential biomarkers and effective therapeutic targets for ACC.

## Introduction

Adenoid cystic carcinoma (ACC) was discovered a long time ago with location in the major and minor salivary glands [1,2] and other organs [3], which can be divided into tubular form (Grade I), cribriform (Grade II) and solid form (Grade III) [4]. Clinical staging had more significant importance than histological grade on prognosis [5]. The 10-year survival rates are approximately 73% (Stage I), 43% (Stage II) and 15% (Stage III and IV) [4]. Studies on oral ACC (OACC) are even more uncommon and insufficient. Surgery and postoperative radiotherapy have been the standard treatment of ACC for a long time [6] with poor prognosis (up to 40% of recurrent rate and 60% of metastatic rate) [7]. Several factors have been studied to associate with the clinicopathological parameters and prognosis of ACC, including p53 [8], SOX2 [9], mutated ATM [10], MACC1 [11], WHSC1 [12], EpCAM [13], PSMA [14], TRAF6 [15], HSP27 [16], PRRX1 [17], hypoxia-related genes [18], the EGFR pathway genes [19], MYB–NFIB fusion genes [20–23], as well as NOTCH1-HEY1 pathway [24] and Akt signaling pathway [25,26]. Considering the aggressive behavior, it is urgent to figure out more efficient clinical pathological and biomolecular prognostic factors for therapeutic choices.

Protein post-translational modifications (PTMs) exist in both eukaryotes and prokaryotes at one or more sites [27]. More than 200 types of PTMs are identified in humans [28]. Ubiquitination is the covalent conjugation in proteins by conserved small protein ubiquitin and ubiquitin-like (Ubl) proteins either as a monomer or as a polyubiquitin chain. [29] Ubiquitin-activating protein E1, ubiquitin-conjugating protein E2 and ubiquitin-ligase E3 are involved to form a transient reaction, which can be reversed by deubiquitylating enzymes (DUBs) [30–32]. The modified substrates are degraded after binding to a multisubunit protease complex [29]. The dynamic changes of ubiquitination form an enzymatic and complete ordered system to control subcellular processes [33], which affects pathophysiological states in cancer under various conditions [34,35]. Both oncogenes and tumor-suppressor genes undergo ubiquitination [36]. Several studies mentioned the potential of ubiquitination related proteins as OACC biomarkers. From Nanostring nCounter miRNA assay, ubiquitin-like modifier activating enzyme 2 (UBA2) was identified increased in primary and recurrent tumors than normal tissue, revealing the potential connection between UBA2 and tumor recurrence and metastasis [37]. The expression of Ubiquitin-specific protease 22 (USP22) in salivary ACC (SACC) was higher in the tumor group than in the adjacent normal group, which was associated with a poor prognosis [38]. The low expression of the tumor suppressor gene cylindromatosis (CYLD), which has deubiquitinating enzyme activity, corrected with salivary gland tumor progression through NF-κB pathway [39]. Notwithstanding, there is lack of relevant research on the molecular mechanisms and related pathogenesis of ubiquitination network in OACC.

A global and comprehensive information about ubiquitin system is difficult due to the challenge for high-throughput analysis [40]. Another obstacle is that these revisable modifications can be easily lost or not easily detected during the experiment. The mass spectrometry (MS)-based proteomic approaches have been widely used in qualitative and quantitative analysis of cellular biology [41]. The organic combination of non-standard quantitative and MS-based proteomic technology can effectively identify ubiquitination substrates and modification network in OACC.

In our project, 4D label-free quantitative ubiquitination proteomics was carried out. A total of 4152 ubiquitination sites were identified on 1993 proteins, in which 1648 loci of 859 proteins contained quantitative information. We also conducted a systematic bioinformatics analysis, including protein annotation, functional classification, functional enrichment and cluster analysis based on functional enrichment proteins. The proteomic methodologies in our work illustrating ubiquitination landscape in OACC can be applied to the search and identification of novel molecular biomarkers, provide valuable information for diagnosis and help discovering novel therapeutic anticancer strategies.

## Materials and methods

### Sample preparation

Four pairs of OACC tumor and adjacent normal tissues were collected from Shenzhen People’s Hospital. The present study was carried out following the Declaration of Helsinki and approved by the Medical Ethics Committee of Shenzhen People’s Hospital (No. LL-KY-2019173). Participants were informed about the introduction of the present study, signed an informed consent, and agreed to take tissue samples after surgical resection for scientific research. Parts of the clinical information of patients are listed in Table 1.

**Table 1:** Clinical information in OACC tumor and adjacent normal tissues

Gender	Age (years)	Tumor size (cm)	Tumor location	Pathological diagnosis	Neuro recidivist	Lymph node metastasis
Female	32	1.5	Right submandibular	ACC	Yes	No
Male	58	3.0	Left submandibular	ACC	Yes	Yes
Female	23	1.0	Palate	ACC	Yes	No
Male	64	2.5	Parotid gland and neck	ACC	Yes	Yes

### Protein extraction

Appropriate number of samples were added with liquid nitrogen to grind to powder and added with four-times of powder lysis buffer (1% Triton X-100, 1% protease inhibitor, 50 μm PR-619, 3 μm TSA, 50 mm NAM) for ultrasonic pyrolysis. After centrifugation in 12000×***g*** for 10 min, the supernatant was transferred to a new centrifuge tube. The protein concentration was determined by BCA kit.

### Trypsin digestion

TCA was slowly added in each sample, followed by precipitating at 4°C. The supernatant was discarded after centrifugation in 4500×***g*** for 5 min. The precipitate was washed with pre-cooled acetone, dried in the air and dissolved by 200 mm TEAB buffer. Trypsin was added and the reaction was maintained overnight. After the incubation by dithiothreitol at 56°C for 30 min, iodoacetamide (IAA) was added. The samples were then incubated at room temperature for 15 min in dark.

### MS

The digested peptides were dissolved by liquid chromatography mobile phase A (containing 0.1% formic acid and 2% acetonitrile) and separated by NanoElute ultra performance liquid chromatography system. The flow rate was 450.00 nl/min, and the gradient of mobile phase B (containing 0.1% formic acid and 100% acetonitrile) was set as follows: 6–22%: 0–43 min; 22–30%: 43–56 min; 30–80%: 56–58 min; 80%: 58–60 min. The peptides were separated by Ultra Performance Liquid Chromatography (UPLC) and injected into Capillary Ion Source for ionization to be analyzed by Tims-TOF Pro MS. The voltage of ion source was 2.0 kV, and the peptide parent ion and its secondary fragments were detected and analyzed by high-resolution TOF. The scanning range of secondary MS is set to 100–1700. The data acquisition mode is PASEF. After the collection of a first-order mass spectrum, the second-order mass spectrum with charge number of parent ion in the range of 0–5 was collected ten-times in PASEF mode, and the dynamic exclusion time of tandem MS scanning was set to 30 s.

### Database searching

Maxquant 1.6.6.0 was used to retrieve the secondary MS data. The proteins were detected in *Homo sapiens* 9606 (20366 sequences) with the common contamination database. The additional reverse database was added to calculate the false discovery rate (FDR) caused by random matching. The FDR and peptide spectrum matches (PSM) was set to 1%. Enzyme digestion: trypsin/P. Number of missed sites: 4. Minimum length: 7. Maximum modification number: 5. Mass error tolerance of primary parent ion: 20.0 ppm in first search and 20 ppm in main search. Mass error tolerance of secondary fragment ion: 20.0 ppm. Cysteine alkylation carbamidomethyl (c) was set as fixed modification, and the variable modification was [‘acetyl (protein N-term)’, ‘oxidation (m)’, ‘glygly (k)’]. The quantitative method was set to LFQ.

## Results

### Systematic profiling of protein ubiquitination in OACC samples

**Figure 1:**
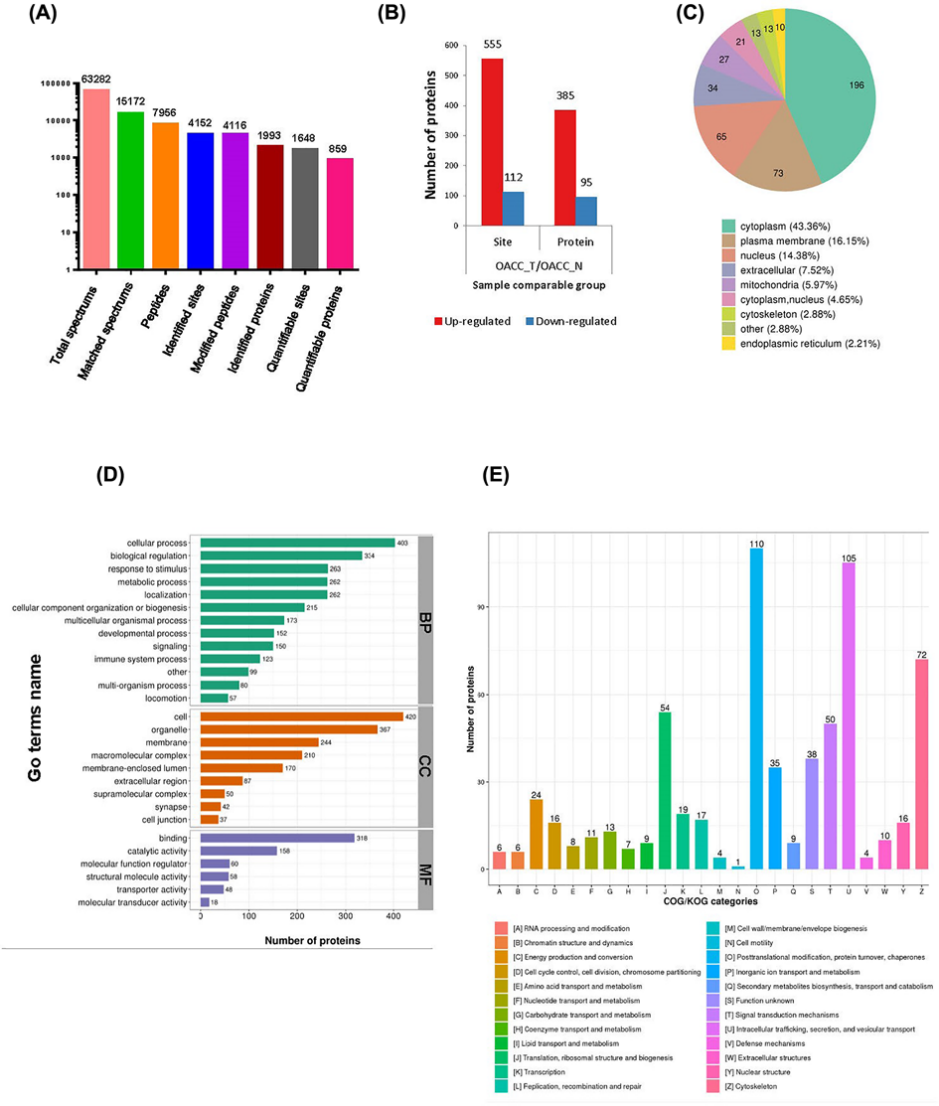
Identification of protein ubiquitination (**A**) Number of proteins and modification sites identified according to MS data. (**B**) Differentially modified sites and proteins in tumor and normal samples, respectively (filtered with threshold value of expression fold change > 1.5 and *P*-value <0.05). (**C**) Subcellular location of differentially ubiquitinated proteins. (**D**) Functional category of differentially ubiquitinated proteins in GO terms. (**E**) COG functional classification of differentially ubiquitinated proteins. Abbreviations: BP, biological process; CC, cellular component; COG, clusters of original groups; GO, Gene Ontology; MF, molecular function.

In order to globally reveal the involvement of ubiquitin in the progression and regulation of OACC, we performed 4D label-free quantitative ubiquitination proteomics study through comparing OACC tumor samples (OACC_T) with the adjacent normal samples (OACC_N) in four patients who had not received any drug treatment before operation. The identification data were filtered as localization probability > 0.75. After MS analysis and database search, a total of 63282 secondary spectra were obtained, of which 15172 were available. The relative quantitative value was obtained according to the intensity of the modified site between different samples. According to this method, 7956 peptides and 4116 modified peptides were identified. Among 4152 ubiquitination sites in 1993 proteins, 1648 sites in 859 proteins were quantified (Figure 1A). OACC_T showed 555 ubiquitination sites up-regulated (≥1.5-fold, *P*-value <0.05) in 385 proteins and 112 ubiquitination sites down-regulated (≤0.67 fold, *P*-value <0.05) in 95 proteins compared with normal samples (Figure 1B). The top 20 proteins and the corresponding sequence were listed in Table 2.

**Table 2:** Top 20 up- and down-regulated proteins and the corresponding sequence (normalized modification sites quantitation)

Protein accession	Gene name	Modified sequence	OACC_T/OACC_N ratio	Regulated type
P62979	*RPS27A*	IQDK(1)EGIPPDQQR	26.637	Up
O43175	*PHGDH*	AWAGSPK(1)GTIQVITQGTSLK	16.252	Up
Q8NBN3	*TMEM87A*	FAFSPLSEEEEEDEQK(0.944)EPMLK(0.056)	11.91	Up
P54725	*RAD23A*	IDEK(1)NFVVVMVTK	8.567	Up
P54727	*RAD23B*	IDEK(1)NFVVVMVTK	8.17	Up
P54727	*RAD23B*	IDIDPEETVK(0.996)ALK(0.004)	8.17	Up
P68363	*TUBA1B*	AYHEQLSVAEITNACFEPANQMVK(1)CDPR	8.154	Up
P06213	*INSR*	GGK(1)GLLPVR	6.307	Up
P62736	*ACTA2*	K(1)DLYANNVLSGGTTMYPGIADR	6.263	Up
P68363	*TUBA1B*	TIGGGDDSFNTFFSETGAGK(1)HVPR	6.208	Up
P62873	*GNB1*	K(1)ACADATLSQITNNIDPVGR	6.169	Up
Q5BJD5	*TMEM41B*	AVK(1)WSQQVER	6.09	Up
P43121	*MCAM*	SDK(1)LPEEMGLLQGSSGDK	6.085	Up
P63261	*ACTG1*	K(1)DLYANTVLSGGTTMYPGIADR	5.928	Up
P08670	*VIM*	ETNLDSLPLVDTHSK(1)R	5.9	Up
Q9Y487	*ATP6V0A2*	VTK(1)TFVK	5.834	Up
P15311	*EZR*	LTPK(1)IGFPWSEIR	5.442	Up
O75881	*CYP7B1*	DDFLK(0.999)FDDK(0.001)	5.421	Up
P83731	*RPL24*	AITGASLADIMAK(1)R	5.359	Up
P55287	*CDH11*	K(1)EPLIVFEEEDVR	5.357	Up
P05023	*ATP1A1*	AAVPDAVGK(1)CR	0.031	Down
P68363	*TUBA1B*	LSVDYGK(0.98)K(0.946)SK(0.074)	0.033	Down
Q13797	*ITGA9*	YK(1)EIIEAEK	0.062	Down
Q8WZ42	*TTN*	ITNYIVEK(1)CATTAER	0.175	Down
Q13797	*ITGA9*	EIIEAEK(1)NR	0.202	Down
Q9HCU0	*CD248*	WVIHAGSK(1)SPTEPMPPR	0.219	Down
P62841	*RPS15*	EAPPMEKPEVVK(1)THLR	0.244	Down
P15104	*GLUL*	K(1)DPNK(1)LVLCEVFK	0.245	Down
Q99808	*SLC29A1*	SLTAVFMWPGK(1)DSR	0.267	Down
P49411	*TUFM*	K(1)YEEIDNAPEER	0.268	Down
Q05086	*UBE3A*	AAAK(1)HLIER	0.268	Down
Q7L1W4	*LRRC8D*	DGEQAK(1)ALFEK	0.291	Down
O43707	*ACTN4*	K(1)HEAFESDLAAHQDR	0.294	Down
P23229	*ITGA6*	EIK(0.003)DEK(0.997)YIDNLEK	0.324	Down
P08133	*ANXA6*	PANDFNPDADAK(1)ALR	0.331	Down
P35555	*FBN1*	GQCIK(1)PLFGAVTK	0.331	Down
Q96D96	*HVCN1*	LK(1)QMNVQLAAK	0.334	Down
P09525	*ANXA4*	ISQK(1)DIEQSIK	0.335	Down
P42167	*TMPO*	YVPLADVK(0.95)SEK(0.05)	0.336	Down
P19388	*POLR2E*	GQVVK(1)IIR	0.343	Down

### Functional classification of differentially ubiquitinated proteins

In order to better study the ubiquitinated proteins in OACC, we firstly investigated the subcellular location of differentially ubiquitinated proteins (DUPs). Nearly half of the ubiquitinated proteins located in cytoplasm (*n*=196, 43.36%), followed by plasma membrane (*n*=73, 16.15%) and nucleus (*n*=65, 14.38%) (Figure 1C). We also performed the up- and down- subcellular localization classification, respectively (Supplementary Figure S1A,B).

The Gene Ontology (GO) knowledgebase, or GO terms, can provide specific definition of protein functions. It contains three kinds of non-overlapping ontologies: biological process (BP), cellular component (CC) and molecular function (MF). A total of 2573 proteins (53%) were analyzed in the BP classification, in which most of the proteins were associated with cellular process, biological regulation and response to stimulus. Within the definition of CC category to 1632 proteins (33%), the DUPs were mainly involved in the cell, organelle and membrane. The MF category of 671 proteins (14%) showed that 318 and 158 proteins were associated with the binding and catalytic activity, respectively (Figure 1D). The GO definition of up- and down-regulated protein were established (Supplementary Figure S1C,D). Clusters of original groups (COG) is also defined as homologous protein cluster. We classified DUPs into COG functional groups, in which the most abundant are PTM protein turnover and chaperones (Figure 1E).

### Functional enrichment analysis of DUPs

**Figure 2:**
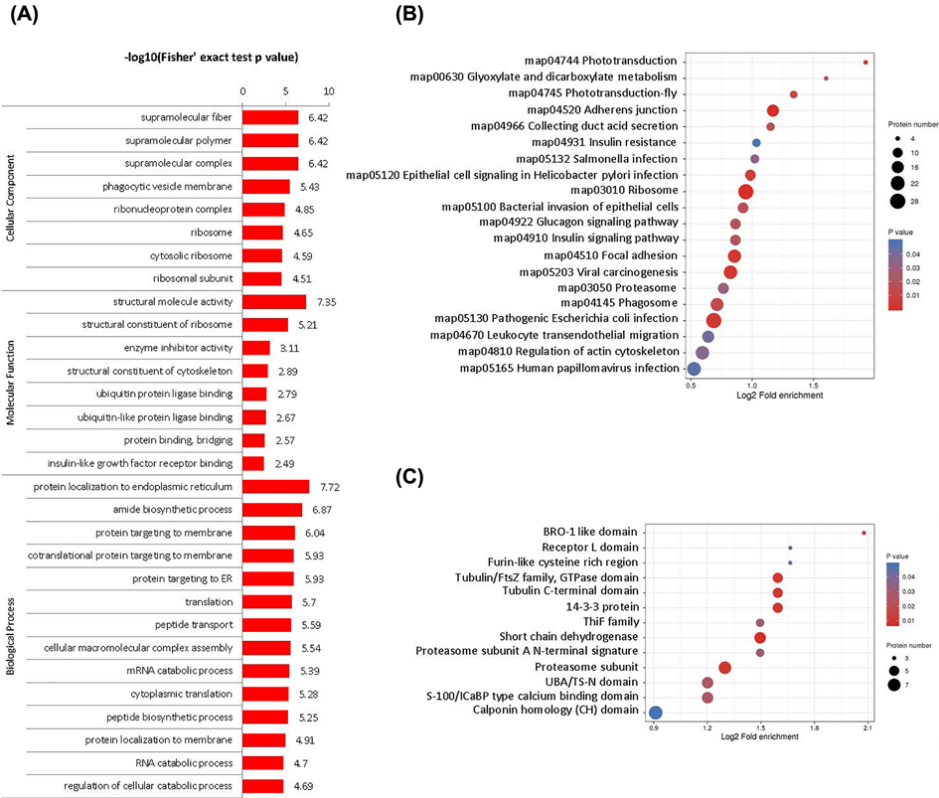
Functional classification of DUPs (**A**) GO enrichment analysis, (**B**) KEGG pathway analysis and (**C**) protein domain enrichment of DUPs. In the bubble chart, the vertical axis is the functional classification or pathway, and the horizontal axis is the log2 converted value of the proportion of different proteins in the functional type compared with the proportion of identification proteins. The circle color indicates the enrichment of significant *P*-value, and the circle size indicates the number of differential proteins in functional class or pathway.

We conducted the functional enrichment analyses with GO annotation and Kyoto Encyclopedia of Genes and Genomes (KEGG) pathway enrichment analysis. Followed by phagocytic vesicle membrane, cytosolic ribosome and ribosomal subunit most differed in CC category. In MF category, the most DUPs were concentrated in insulin-like growth factor receptor binding, structural constituent of ribosome and structural constituent of cytoskeleton. Meanwhile, cytoplasmic translation, protein localization to endoplasmic reticulum, co-translational protein targeting membrane and protein targeting ER were most altered in BP category (Figure 2A; Supplementary Table S1).

According to the KEGG pathway analysis, we identified 25 pathways from up-regulated and 10 pathways from up-regulated DUPs. The most significantly up-regulated were phototransduction (map04744), glyoxylate and dicarboxylate metabolism (map00630), and phototransduction-fly (map04745). Meanwhile, aldosterone-regulated sodium reabsorption (map04960), fatty acid elongation (map00062) and terpenoid backbone biosynthesis (map00900) were the mostly down-regulated (Figure 2B; Supplementary Table S2). Protein domain is the unit of protein evolution, which has similar sequence, structure and function. We performed enrichment analysis on the domain level (Figure 2C). Regarding the BRO1-like domain, the most significantly up-regulated domains contained ALIX V-shaped domain binding to HIV, regulated-SNARE-like domain and XPC-binding domain. Furthermore, PLD-like domain was the most significantly down-regulated domain (Supplementary Table S3).

### Cluster analysis of differential modified proteins

**Figure 3:**
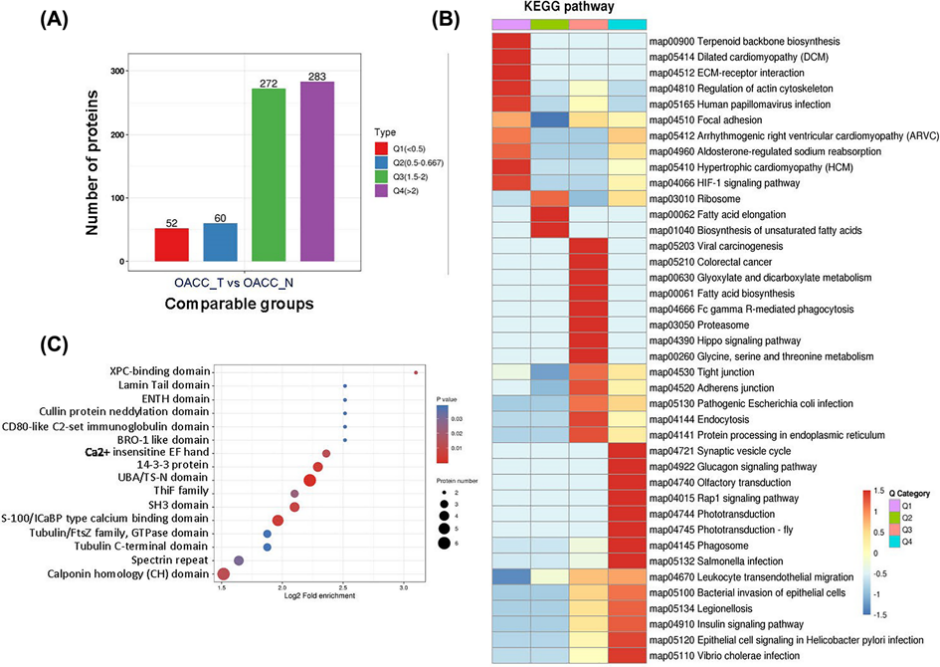
Cluster analysis of differential modified proteins (**A**) Protein number in each cluster. (**B**) KEGG pathway enrichment analysis of all clusters by heatmap. The color blocks corresponding to the functional description of the differentially expressed proteins in different groups indicated the degree of enrichment. Red indicates strong enrichment and blue indicates weak enrichment. (**C**) Protein domain enrichment analysis in Q3 cluster.

In order to test the rationality and accuracy of the identified Figure 2B, we used cluster analysis to aggregate the proteins according to the trend of expression. In order to find the correlation of protein functions in different fold change, we divide DUPs into four clusters according to OACC_T/OACC_N ratio, which were called Q1 (ratio < 0.5), Q2 (ratio between 0.5 and 0.667), Q3 (ratio between 1.5 and 2) and Q4 (ratio > 2) (Figure 3A). In GO enrichment analysis, chromaffin granule membrane, cytosolic small ribosomal subunit, myelin sheath and polysome differed the most in each cluster by CC category. In MF category of each cluster, the most enriched were acid-ammonia (or amide) ligase activity, neurexin family protein binding, ion channel binding and ADP binding. In addition, filopodium assembly, mitochondrial RNA metabolic process, positive regulation of stress fiber assembly and sarcomere organization were the top regulated in BP category in each cluster (Supplementary Table S4). According to the *P*-value in Fisher’s exact test obtained by enrichment analysis, hierarchical clustering method is used to gather the related functions in different groups and draw a Heatmap. Regarding KEGG analysis, terpenoid backbone biosynthesis (map00900), fatty acid elongation (map00062), glyoxylate and dicarboxylate metabolism (map00630), and phototransduction (map04744) enriched most in each cluster (Figure 3B; Supplementary Table S5). XPC-binding domain, BRO1-like domain, CD80-like C2-set immunoglobulin domain, cullin protein neddylation domain, ENTH domain and lamin tail domain were mostly enriched in Q3 cluster according to protein domain enrichment analysis (Figure 3C; Supplementary Table S6).

### Protein–protein interaction network of differential modified proteins

**Figure 4:**
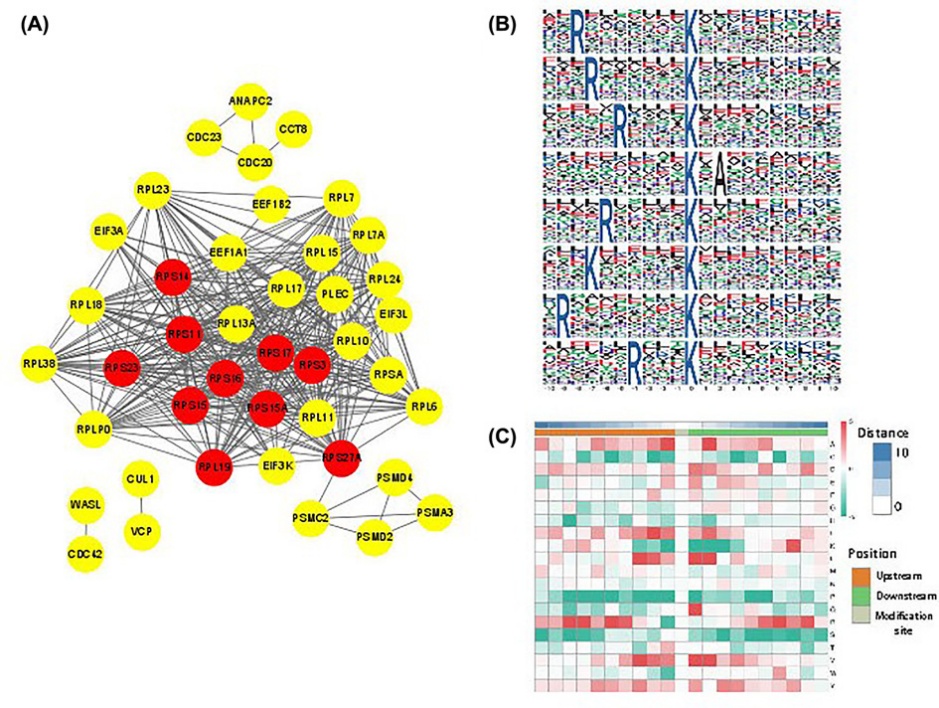
PPI network and Motif analysis of ubiquitination sites (**A**) PPI network analyses of differentially expressed ubiquitinated proteins analyzed by STRING database. The red circles marked top ten hub proteins based on degree value analyzed by software Cytoscape. (**B**) Significantly enriched ubiquitination motifs by Motif-X. (**C**) Motif enrichment heat map of ubiquitination. Red indicates that the amino acid is significantly enriched near the ubiquitination sites, and green indicates that the amino acid is significantly reduced near the ubiquitination sites.

Protein–protein interaction (PPI) network is composed of proteins through their interactions. In the network diagram, nodes represent proteins, and nodes are labeled with the names of these proteins. The interaction between two proteins is connected by wires. According to the protein interaction database of STRING (v.10.5) [42], we performed PPI network by selecting the top 50 proteins based on degree. The interaction relationship of DUPs was extracted according to the confidence score > 0.7 (high confidence) (Figure 4A; Supplementary Table S7). According to analysis by software Cytoscape, the top ten hub proteins were listed (Table 3).

**Table 3:** Top ten hub proteins in PPI network based on degree value

Name	Degree	Betweenness centrality	Closeness centrality	Neighborhood connectivity	Clustering coefficient
RPS16	28	0.021698041	0.804878049	23.21428571	0.82010582
RPS11	28	0.021698041	0.804878049	23.21428571	0.82010582
RPS3	28	0.021698041	0.804878049	23.21428571	0.82010582
RPS17	28	0.021698041	0.804878049	23.21428571	0.82010582
RPS15A	27	0.020608616	0.785714286	23.2962963	0.823361823
RPS15	27	0.017387074	0.76744186	23.48148148	0.84045584
RPS27A	26	0.222127935	0.825	24.15384615	0.886153846
RPS23	26	0.00803387	0.76744186	24.38461538	0.907692308
RPS14	26	0.010250981	0.75	24.11538462	0.886153846
RPL19	25	0.002430965	0.75	25	0.96

### Motif analysis of protein modification

Protein motif analysis calculates the regular trend of amino acid sequences in the region of modification sites. This kind of analysis can find the sequence characteristics, so as to speculate or determine the modification-related enzymes. We identified eight conserved ubiquitination motifs analyzed by Motif-X (Figure 4B). The enrichment of specific amino acids neighboring the ubiquitination sites were exhibited (Figure 4C).

## Discussion

ACC of the salivary glands performs the properties of slow-growing, local and/or distant spread, nodal positivity and high mortality, as well as high rate of occurrence and metastasis [43,44]. Several specific prognostic factors had been identified the association with ACC [45]. Meanwhile, most of the related studies are based on the statistics and correlation analysis of clinical cases, and lack of more detailed and in-depth biological mechanism research on the occurrence and progress of the disease. The ubiquitination modification plays a significant role in cancer pathology [46]. MS can be used to analyze the role of protein PTMs in human diseases, and PTM-based protein variants can be explored as deeply as possible.

Ribosomal protein S27a (RPS27A) is the top differentially up-regulated protein in our identification. RPS27A performs multifunction in ribosome biogenesis and protein PTMs, contributing to progression of leukemia or solid tumors [47,48]. A ubiquitin-fused RPS27A protein (Uba80) was reported related to apoptotic cell death and overexpressed in colon and renal cancer [49–52]. However, the molecular mechanism of RPS27A-related ubiquitination in tumors remains to be studied. Phosphoglycerate dehydrogenase (PHGDH) is the rate-limiting enzyme of *de novo* serine biosynthesis pathway, which is closely related to the occurrence and development of many kinds of tumors [53,54]. The serine synthesis during cancer progression was suppressed when PHGDH went through Parkin-related ubiquitination and degradation [55,56]. PHGDH is also a ubiquitination substrate of RNF5 in the study of breast cancer cells [57]. The PHGDH ubiquitination in OACC has not been reported. TMEM87A, also named as Elkin1, is important in cell–cell adhesin and metastasis with limited studies [58]. The insulin receptor (INSR) is a key regulator in metabolic homeostasis through diverse signal pathways including PI3K/AKT and MAPK [59]. Although phosphorylation is critical in INSR-dependent signal cascade, the ubiquitin/proteasome system modulate degradation of transducers in this pathway [60,61]. The biological function of these targets in OACC and their combination with metabolic abnormalities and immune regulation will drive us to further study how gene changes modulate the behavior of cancer cells, so as to unlock more effective treatment methods.

In addition, ATP1A1, Tubulin α1b (TUBA1B) and integrin subunit α9 (ITGA9) are the top down-regulated according to OACC_T/OACC_N ratio. As a membrane-bound ion pump, Na^+^/K^+^-ATPase shows tissue-specific profile [62]. The overexpression of α1 subunit of Na^+^/K^+^-ATPase (ATP1A1) were observed in esophageal squamous cell carcinoma [63], non-small-cell lung cancer and hepatocellular carcinoma, contributing to cancer proliferation and migration [64,65]. However, ATP1A1 were significantly down-regulated in prostate cancer [66,67], colorectal cancer [68] and renal cell carcinoma [69]. The function and PTM regulation of ATP1A1 in OACC is not yet fully clear. TUBA1B belongs to cytoskeleton compartment with a central function in cell shape maintenance and cellular process regulation, especially in cell division [70]. The higher expression of TUBA1B and poor prognosis were reported in hepatocellular carcinoma [71]. Interesting, the ubiquitination at different sites of TUBA1B shows different up- and down-regulation trends in tumor tissues (Table 2). This suggests that TUBA1B ubiquitination regulation may have different biological functions. With the continuous deepening of cytoskeleton-related research, the importance of microtubules in tumor metastasis has begun to become prominent. The ITGA9 belongs to integrin protein family and a partner of β1 subunit facilitating the interaction of cell–cell and cell–extracellular matrix [72]. Depletion of ITGA9 suppressed breast cancer progression and metastasis through GSK3/β-catenin pathway [73]. The decreased expression of ITGA9 in lung cancer indicated potential genetic and epigenetic regulation mechanism [74]. Besides TUBA1B and ITGA9, other cell cytoskeleton and cell adhesion associated proteins were also identified in our results, including ACTA2, MCAM, ACTG1, VIM and CDH11. Due to the high recurrence and metastasis of OACC, research on the modification of cytoskeleton and cell adhesion will help to further clarify the mechanism of OACC metastasis.

Considering the hub genes of interaction regulation by Cytoscape analysis, we found they all belong to ribosomal proteins. Ribosomal proteins are abnormally expressed in a variety of tumors, which affect the apoptosis, aging, growth, invasion, drug and radiation resistance [75]. The expression level of ribosomal protein has become a potential indicator of tumor diagnosis, treatment and prognosis [76]. Through the in-depth study of abnormal expression and ubiquitination modification of ribosomal proteins in tumor tissue, we can further understand the role of high expression of ribosomal protein gene in malignant tumor.

Protein ubiquitination plays a very important role in cellular processes such as subcellular localization, growth, apoptosis and metabolism. The ubiquitination modification omics based on MS has been used for disease biomarkers and pathogenic mechanism analysis [77,78]. In this project, we studied the ubiquitin proteomics of OACC tumor and adjacent normal tissues, identified the different ubiquitin modification sites, analyzed the function of the identified ubiquitinated proteins, and obtained eight protein clusters. Our work enriched the scope of OACC research and became a reference for the development of novel targets. These results also supported the possible functions of the ubiquitination of cell skeleton and extracellular matrix associated proteins in OACC development and metastasis. However, whether the selected differential protein can be used as a therapeutic target for OACC still needs more detailed *in vivo* and *in vitro* experiments to verify, so as to better carry out precise treatment for OACC, add further knowledge specifically for patients and explore more promising combination therapy.

## Supplementary Material

Supplementary Figure S1 and Tables S1-S7

## Data Availability

All data included in the present study are available upon request by contact with the corresponding authors.

## Abbreviations

ACC: adenoid cystic carcinoma
BP: biological process
CC: cellular component
COG: clusters of original groups
CYLD: cylindromatosis
DUP: differentially ubiquitinated protein
ER: Endoplasmic Reticulum
FDR: false discovery rate
GO: Gene Ontology
IAA: iodoacetamide
INSR: insulin receptor
ITGA9: integrin subunit α9
KEGG: Kyoto Encyclopedia of Genes and Genomes
MF: molecular function
MS: mass spectrometry
OACC: oral ACC
PHGDH: phosphoglycerate dehydrogenase
PPI: protein–protein interaction
PSM: peptide-spectrum matches
PTM: post-translational modification
RPS27A: ribosomal protein S27a
SACC: salivary adenoid cystic carcinoma
TCA: Trichloroaceticacid
TUBA1B: Tubulin α1b
UBA2: ubiquitin-like modifier activating enzyme 2
UPLC: Ultra Performance Liquid Chromatography
USP22: Ubiquitin-specific protease 22
