# Supplementary material for: Quantitative ubiquitylomics reveals the ubiquitination regulation landscape in oral adenoid cystic carcinoma
Source: Biosci Rep. 2021 Aug 20;41(8):BSR20211532. doi: 10.1042/BSR20211532 (PMC8385350; doi:10.1042/BSR20211532)
Supplement: Supplementary Figure S1 and Tables S1-S7 [file BSR-2021-1532_supp.pdf]

Supplementary material

for

Quantitative ubiquitylomics reveals the ubiquitination regulation landscape in oral adenoid cystic carcinoma

Wen Li [1,2,3], Xiaobin Wang[1,2,3], Qian Zhang[1,2,3], Hanlin Wang[3], Wenxin Zuo[4], Hongliang Xie[4], Jianming Tang[4], Mengmeng Wang[4], Zhipeng Zeng[4], Wanxia Cai[4], Donge Tang[4*], Yong Dai[4*]

[1] Carson International Cancer Centre, Shenzhen University General Hospital and Shenzhen University Clinical Medical Academy Centre, Shenzhen University, 1098 Xueyuan Road, Shenzhen Guangdong 518000, China

[2] Key Laboratory of Optoelectronic Devices and Systems, College of Physics and Optoelectronic Engineering, Shenzhen University, Shenzhen 518060, China

[3] Health Science Center, School of Medicine, Shenzhen University, Shenzhen 518060, China

[4] Clinical Medical Research Center, Guangdong Provincial Engineering Research Center of Autoimmune Disease Precision Medicine, Shenzhen Engineering Research Center of Autoimmune Disease, The Second Clinical Medical College of Jinan University, The First Affiliated Hospital of Southern University of Science and Technology, Shenzhen People’s Hospital, Shenzhen, Guangdong 518020, China

[*]To Whom correspondence should be addressed:

Donger Tang, Tel: +86-0755-22942106; Email: donge66@126.com.

Yong Dai, Tel: +86-0755-22942780; Email: daiyong22@aliyun.com.

Postal address: 1017 Dongmen North Road, Luohu District, Shenzhen 518020, Guangdong Province, China

Supplementary material content

Table SI. Go enrichment analyses S4

Table SII. KEGG pathway enrichment S7

Table SIII. Protein domain enrichment S9

Table SIV. GO enrichment in each cluster S10

Table SV. KEGG enrichment in each cluster S13

Table SVI. Protein domain enrichment in each cluster S15

Table SVII. Interaction network S16

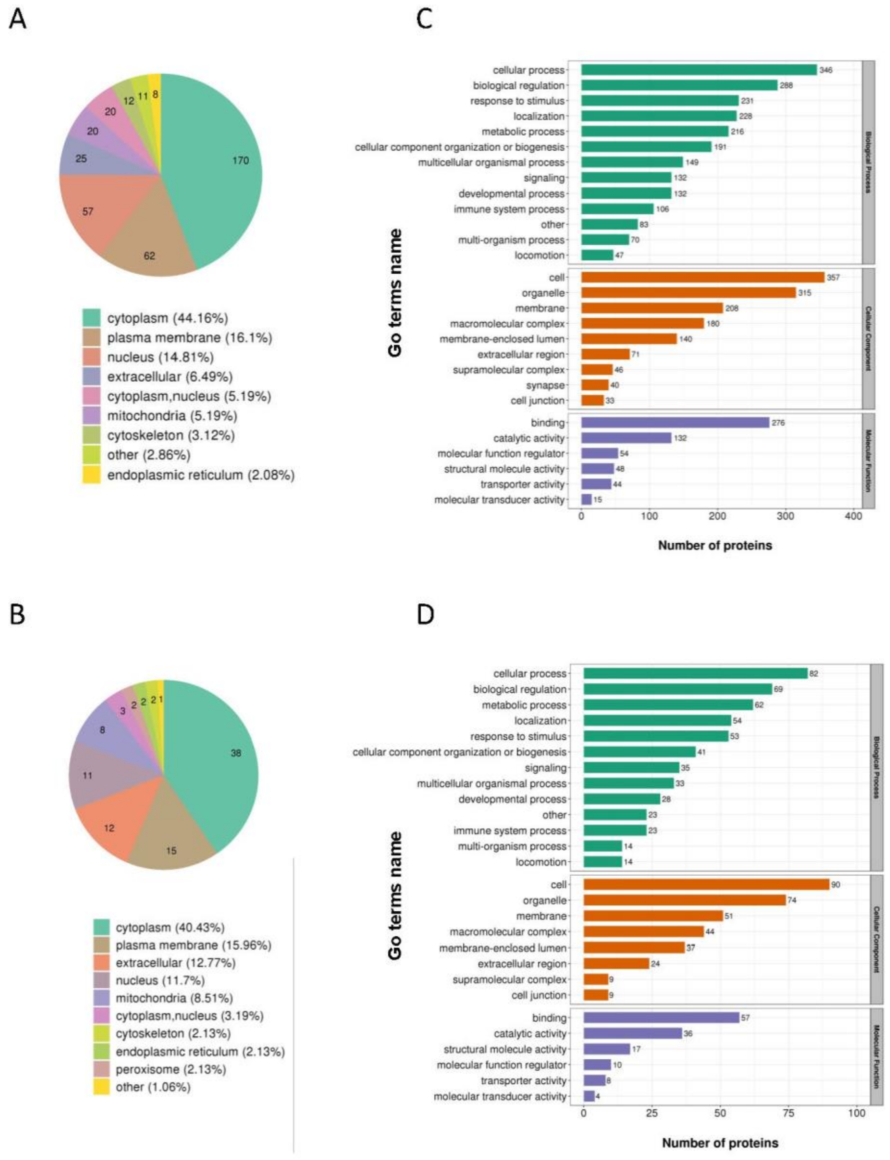

Figure S1. Functional classification of up- and down-regulated proteins. (a) Up-regulated and (b) down-regulated subcellular localization classification. The GO definition of (c) up-regulated and (d) down-regulated proteins.

Table SI. Go enrichment analyses

	GO Terms	GO Terms Description	Mapping	Background	Fold Enrichment	-log10(Fisher's exact test p value)
Up	Cellular Component	supramolecular complex	46	114	2.08	6.95
		supramolecular polymer	46	114	2.08	6.95
		supramolecular fiber	46	114	2.08	6.95
		phagocytic vesicle membrane	12	17	3.64	5.24
		cytoskeletal part	61	193	1.63	4.73
		secretory granule	71	239	1.53	4.47
		intercalated disc	12	20	3.1	4.18
		polysome	12	20	3.1	4.18
	Molecular Function	structural molecule activity	48	129	1.83	5.44
		ubiquitin-like protein ligase binding	37	106	1.71	3.57
		ubiquitin protein ligase binding	36	101	1.75	3.55
		amino acid binding	6	8	3.68	2.88
		protein binding, bridging	13	28	2.28	2.81
		ion channel binding	13	28	2.28	2.81
		insulin-like growth factor receptor binding	4	4	4.91	2.77
		structural constituent of ribosome	19	48	1.94	2.76
	Biological Process	peptide transport	80	250	1.6	6.01
		cellular macromolecular complex assembly	46	125	1.84	5.16
		amide transport	80	259	1.55	5.15
		regulation of cellular catabolic process	55	166	1.66	4.61
		protein localization to endoplasmic reticulum	24	55	2.19	4.33
		alpha-amino acid catabolic process	6	6	5.01	4.21
		dendrite morphogenesis	9	13	3.47	3.81
		protein targeting to ER	21	49	2.15	3.71
		cotranslational protein targeting to membrane	21	49	2.15	3.71
		regulation of protein localization to membrane	20	46	2.18	3.63
		protein targeting to membrane	23	56	2.06	3.62
		regulation of protein catabolic process	33	92	1.8	3.61
		protein targeting	33	95	1.74	3.29
		neuron projection morphogenesis	24	62	1.94	3.28
Down	Cellular Component	ribosome	11	55	4.07	4.36
		cytosolic ribosome	9	44	4.17	3.72

		extracellular matrix	8	36	4.53	3.62
		rough endoplasmic reticulum	7	29	4.92	3.45
		ribosomal subunit	9	48	3.82	3.42
		cytosolic small ribosomal subunit	6	24	5.09	3.11
		small ribosomal subunit	6	26	4.7	2.91
		extracellular region part	18	174	2.11	2.83
	**Molecular Function**	heparin binding	6	14	8.52	4.53
		structural molecule activity	17	129	2.62	3.9
		glycosaminoglycan binding	6	20	5.97	3.53
		structural constituent of ribosome	9	48	3.73	3.35
		sulfur compound binding	6	25	4.77	2.96
		calcium-dependent phospholipid binding	4	12	6.63	2.67
		phospholipase inhibitor activity	3	6	9.95	2.66
		acid-ammonia (or amide) ligase activity	2	2	19.89	2.6
	**Biological Process**	amide biosynthetic process	20	113	3.63	6.83
		translation	16	93	3.52	5.32
		peptide biosynthetic process	16	96	3.41	5.13
		protein localization to endoplasmic reticulum	11	55	4.1	4.39
		mRNA catabolic process	11	62	3.64	3.88
		RNA catabolic process	11	66	3.41	3.63
		protein targeting to membrane	10	56	3.66	3.59
		cotranslational protein targeting to membrane	9	49	3.76	3.37
		protein targeting to ER	9	49	3.76	3.37
		mRNA metabolic process	13	98	2.72	3.19
		ncRNA metabolic process	9	56	3.29	2.93
		cytoplasmic translation	6	26	4.73	2.92
		cellular response to interleukin-12	5	19	5.39	2.78
		interleukin-12-mediated signaling pathway	5	19	5.39	2.78
**All**	**Cellular Component**	supramolecular fiber	50	114	1.92	6.42
		supramolecular polymer	50	114	1.92	6.42
		supramolecular complex	50	114	1.92	6.42
		phagocytic vesicle membrane	13	17	3.35	5.43
		ribonucleoprotein complex	48	120	1.75	4.85
		ribosome	27	55	2.15	4.65
		cytosolic ribosome	23	44	2.29	4.59
		ribosomal subunit	24	48	2.19	4.51
	**Molecular Function**	structural molecule activity	58	129	1.88	7.35
		structural constituent of ribosome	26	48	2.26	5.21
		enzyme inhibitor activity	27	63	1.79	3.11

	structural constituent of cytoskeleton	13	24	2.26	2.89
	ubiquitin protein ligase binding	38	101	1.57	2.79
	ubiquitin-like protein ligase binding	39	106	1.53	2.67
	protein binding, bridging	14	28	2.09	2.57
	insulin-like growth factor receptor binding	4	4	4.17	2.49
**Biological Process**	protein localization to endoplasmic reticulum	32	55	2.49	7.72
	amide biosynthetic process	51	113	1.93	6.87
	protein targeting to membrane	30	56	2.29	6.04
	cotranslational protein targeting to membrane	27	49	2.35	5.93
	protein targeting to ER	27	49	2.35	5.93
	translation	42	93	1.93	5.7
	peptide transport	89	250	1.52	5.59
	cellular macromolecular complex assembly	52	125	1.78	5.54
	mRNA catabolic process	31	62	2.14	5.39
	cytoplasmic translation	17	26	2.79	5.28
	peptide biosynthetic process	42	96	1.87	5.25
	protein localization to membrane	50	123	1.74	4.91
	RNA catabolic process	31	66	2.01	4.7
	regulation of cellular catabolic process	62	166	1.6	4.69

Table SII. KEGG pathway enrichment

	KEGG pathway	Mapping	Background	Fold Enrichment	-log10(Fisher's exact test p value)
**Up**	map04520 Adherens junction	15	27	2.42	3.68
	map05203 Viral carcinogenesis	20	45	1.94	2.79
	map05130 Pathogenic Escherichia coli infection	26	66	1.72	2.65
	map04744 Phototransduction	4	4	4.36	2.56
	map04145 Phagosome	19	44	1.88	2.56
	map05100 Bacterial invasion of epithelial cells	12	24	2.18	2.31
	map04910 Insulin signaling pathway	12	25	2.09	2.21
	map04745 Phototransduction - fly	6	9	2.91	2.21
	map04966 Collecting duct acid secretion	7	12	2.54	2.08
	map04510 Focal adhesion	17	42	1.76	2.06
	map05120 Epithelial cell signaling in Helicobacter pylori infection	11	23	2.08	2.03
	map05132 Salmonella infection	8	15	2.32	2.01
	map00630 Glyoxylate and dicarboxylate metabolism	4	5	3.49	1.95
	map04931 Insulin resistance	7	13	2.35	1.83
	map04922 Glucagon signaling pathway	11	25	1.92	1.79
	map03010 Ribosome	20	55	1.58	1.68
	map05110 Vibrio cholerae infection	9	19	2.06	1.65
	map04130 SNARE interactions in vesicular transport	7	14	2.18	1.62
	map03050 Proteasome	12	29	1.8	1.62
	map04611 Platelet activation	14	36	1.69	1.57
	map04670 Leukocyte transendothelial migration	15	39	1.68	1.51
	map04015 Rap1 signaling pathway	17	47	1.58	1.47
	map05134 Legionellosis	6	12	2.18	1.44
	map04721 Synaptic vesicle cycle	8	18	1.94	1.36
	map04970 Salivary secretion	12	31	1.69	1.31
**Down**	map03010 Ribosome	11	55	3.98	4.37
	map04960 Aldosterone-regulated sodium reabsorption	3	9	6.64	2.09
	map04810 Regulation of actin cytoskeleton	7	53	2.63	1.85
	map05410 Hypertrophic cardiomyopathy (HCM)	4	21	3.79	1.74
	map00062 Fatty acid elongation	2	6	6.64	1.49
	map00900 Terpenoid backbone biosynthesis	2	6	6.64	1.49
	map00140 Steroid hormone biosynthesis	2	7	5.69	1.35
	map00565 Ether lipid metabolism	2	7	5.69	1.35
	map01040 Biosynthesis of unsaturated fatty acids	2	7	5.69	1.35
	map04512 ECM-receptor interaction	3	17	3.51	1.31
**All**	map03010 Ribosome	28	55	1.93	4.19
	map04520 Adherens junction	16	27	2.25	3.58

	map05203 Viral carcinogenesis	21	45	1.77	2.54
	map04510 Focal adhesion	20	42	1.81	2.45
	map05130 Pathogenic Escherichia coli infection	28	66	1.61	2.44
	map04744 Phototransduction	4	4	3.8	2.33
	map05120 Epithelial cell signaling in Helicobacter pylori infection	12	23	1.98	2.12
	map04745 Phototransduction - fly	6	9	2.53	1.9
	map04145 Phagosome	19	44	1.64	1.86
	map05100 Bacterial invasion of epithelial cells	12	24	1.9	1.79
	map04966 Collecting duct acid secretion	7	12	2.22	1.73
	map00630 Glyoxylate and dicarboxylate metabolism	4	5	3.04	1.73
	map04910 Insulin signaling pathway	12	25	1.82	1.7
	map04922 Glucagon signaling pathway	12	25	1.82	1.7
	map03050 Proteasome	13	29	1.7	1.51
	map05132 Salmonella infection	8	15	2.03	1.49
	map04810 Regulation of actin cytoskeleton	21	53	1.51	1.44
	map04670 Leukocyte transendothelial migration	16	39	1.56	1.38
	map04931 Insulin resistance	7	13	2.05	1.3

Table SIII. Protein domain enrichment

	Domain description	Mapping	Background	Fold Enrichment	-log10(Fisher's exact test p value)
**Up**	14-3-3 protein	5	7	3.48	2.29
	Tubulin C-terminal domain	5	7	3.48	2.29
	Tubulin/FtsZ family, GTPase domain	5	7	3.48	2.29
	BRO1-like domain	3	3	4.88	2.07
	S-100/ICaBP type calcium binding domain	6	11	2.66	1.89
	UBA/TS-N domain	6	11	2.66	1.89
	Calponin homology (CH) domain	8	18	2.17	1.74
	Proteasome subunit A N-terminal signature	4	6	3.25	1.74
	ThiF family	4	6	3.25	1.74
	Proteasome subunit	6	12	2.44	1.67
	ALIX V-shaped domain binding to HIV	2	2	4.88	1.38
	Regulated-SNARE-like domain	2	2	4.88	1.38
	XPC-binding domain	2	2	4.88	1.38
**Down**	PLD-like domain	2	3	14.4	2.21
	short chain dehydrogenase	3	9	7.2	2.19
	PDZ domain (Also known as DHR or GLGF)	2	8	5.4	1.31
**All**	short chain dehydrogenase	6	9	2.82	2.14
	14-3-3 protein	5	7	3.02	2.01
	Tubulin C-terminal domain	5	7	3.02	2.01
	Tubulin/FtsZ family, GTPase domain	5	7	3.02	2.01
	Proteasome subunit	7	12	2.46	2
	BRO1-like domain	3	3	4.22	1.88
	S-100/ICaBP type calcium binding domain	6	11	2.3	1.59
	UBA/TS-N domain	6	11	2.3	1.59
	Proteasome subunit A N-terminal signature	4	6	2.82	1.51
	ThiF family	4	6	2.82	1.51
	Furin-like cysteine rich region	3	4	3.17	1.36
	Receptor L domain	3	4	3.17	1.36
	Calponin homology (CH) domain	8	18	1.88	1.31

Table SIV. GO enrichment in each cluster

Cluster	GO Terms	GO Terms Description	Fold Enrichment	-log10(Fisher's exact test p value)
Q1	**Cellular Component**	chromaffin granule membrane	41.18	3.24
		A band	13.73	2.99
		sarcomere	5.88	2.9
		basolateral plasma membrane	4	2.86
		integrin complex	12.35	2.85
		protein complex involved in cell adhesion	12.35	2.85
		chromaffin granule	27.45	2.77
		I band	6.86	2.65
	**Molecular Function**	heparin binding	11.1	3.5
		acid-ammonia (or amide) ligase activity	38.83	3.19
		steroid binding	7.77	2.86
		glycosaminoglycan binding	7.77	2.86
		cell adhesion molecule binding	4.4	2.72
		chondroitin sulfate binding	25.89	2.72
		calcium-dependent phospholipid binding	9.71	2.53
		sulfur compound binding	6.21	2.49
	**Biological Process**	amide biosynthetic process	3.34	3
		filopodium assembly	20.97	2.49
		negative regulation of cell development	4.46	2.35
		regulation of cardiocyte differentiation	16.77	2.27
		translation	3.16	2.27
		peptide biosynthetic process	3.06	2.19
		response to cadmium ion	7.4	2.17
		cellular response to metal ion	4.93	2.11
		hemidesmosome assembly	13.98	2.11
		striated muscle contraction	6.29	1.96
		negative regulation of cell differentiation	3.03	1.91
		platelet degranulation	4.19	1.86
		regulation of muscle system process	4.09	1.82
		cellular response to lipid	2.86	1.79
Q2	**Cellular Component**	ribosome	5.72	4.83
		cytosolic ribosome	5.56	3.75
		ribosomal subunit	5.1	3.51
		cytosolic part	3.54	2.85
		ribonucleoprotein complex	2.91	2.79
		extracellular region part	2.41	2.56
		extracellular matrix	4.86	2.52
		cytosolic small ribosomal subunit	5.83	2.38

	**Molecular Function**	structural constituent of ribosome	5.17	3.56
		structural molecule activity	3.02	3.22
		neurexin family protein binding	23.64	2.64
		heparin binding	7.6	2.21
		enzyme inhibitor activity	3.38	2.14
		mRNA 5'-UTR binding	11.82	1.96
		phospholipase inhibitor activity	11.82	1.96
		binding, bridging	4.43	1.95
	**Biological Process**	amide biosynthetic process	4.3	5.82
		protein localization to endoplasmic reticulum	6.3	5.75
		protein targeting to membrane	5.57	4.74
		translation	4.1	4.4
		mRNA catabolic process	5.03	4.37
		cotranslational protein targeting to membrane	5.66	4.3
		protein targeting to ER	5.66	4.3
		peptide biosynthetic process	3.97	4.27
		RNA catabolic process	4.73	4.15
		mRNA metabolic process	3.54	3.48
		negative regulation of wound healing	9.91	3.3
		protein localization to membrane	3.1	3.28
		mitochondrial RNA metabolic process	34.67	3.09
		ncRNA metabolic process	4.33	3.07
**Q3**	**Cellular Component**	myelin sheath	2.59	4.72
		cytoplasmic vesicle part	1.58	4.66
		perinuclear region of cytoplasm	2.03	4.65
		cytoskeletal part	1.86	4.45
		supramolecular complex	2.15	4.33
		supramolecular polymer	2.15	4.33
		supramolecular fiber	2.15	4.33
		secretory granule	1.72	4.14
	**Molecular Function**	enzyme binding	1.63	7.23
		ion channel binding	3.88	5.29
		ubiquitin-like protein ligase binding	2.21	4.73
		ubiquitin protein ligase binding	2.24	4.66
		channel regulator activity	3.19	2.73
		molecular function regulator	1.61	2.73
		double-stranded RNA binding	3.08	2.35
		structural constituent of cytoskeleton	2.79	2.31
	**Biological Process**	regulation of cellular catabolic process	1.84	3.9
		neutrophil activation	1.8	3.77
		regulated exocytosis	1.7	3.72
		neutrophil mediated immunity	1.78	3.7

		positive regulation of organelle organization	1.95	3.49
		regulation of protein catabolic process	2.12	3.48
		regulation of cytoskeleton organization	2.17	3.47
		positive regulation of protein catabolic process	2.49	3.32
		regulation of protein localization to membrane	2.58	3.3
		regulation of organelle organization	1.67	3.2
		striated muscle tissue development	2.75	3.17
		positive regulation of stress fiber assembly	4.62	3.15
		regulation of supramolecular fiber organization	2.4	3.12
		apical junction assembly	3.95	3.09
**Q4**	**Cellular Component**	polysome	5.1	5.9
		ribosome	2.86	4.51
		supramolecular complex	2.19	4.24
		supramolecular fiber	2.19	4.24
		supramolecular polymer	2.19	4.24
		ribosomal subunit	2.9	4.09
		cytosolic ribosome	2.95	3.95
		polysomal ribosome	4.63	3.56
	**Molecular Function**	structural molecule activity	2.42	7.13
		structural constituent of ribosome	2.89	4.38
		binding, bridging	3.25	3.96
		protein binding, bridging	3.41	3.88
		protein domain specific binding	1.89	3.41
		oxidoreductase activity, acting on NAD(P)H	4	2.73
		ADP binding	3.72	2.53
		carboxylic acid binding	2.69	2.42
	**Biological Process**	protein targeting	2.66	6.44
		peptide transport	1.91	6.41
		protein targeting to membrane	3.22	6.17
		cotranslational protein targeting to membrane	3.31	5.8
		protein targeting to ER	3.31	5.8
		amide transport	1.84	5.78
		protein localization to endoplasmic reticulum	2.95	4.97
		mRNA catabolic process	2.62	4.18
		regulation of protein catabolic process	2.25	3.99
		regulation of cellular protein catabolic process	2.54	3.98
		protein localization to membrane	2.05	3.87
		RNA catabolic process	2.46	3.79
		sarcomere organization	6.44	3.55
		striated muscle tissue development	2.92	3.43

Table SV. KEGG enrichment in each cluster

Cluster	KEGG pathway	Mapping	Background	Fold Enrichment	-log10(Fisher's exact test p value)
Q1	map05410 Hypertrophic cardiomyopathy (HCM)	4	21	6.88	2.68
	map04810 Regulation of actin cytoskeleton	6	53	4.09	2.6
	map04510 Focal adhesion	5	42	4.3	2.31
	map05414 Dilated cardiomyopathy (DCM)	4	30	4.81	2.1
	map04512 ECM-receptor interaction	3	17	6.37	2
	map00900 Terpenoid backbone biosynthesis	2	6	12.03	1.98
	map05165 Human papillomavirus infection	5	58	3.11	1.72
	map05412 Arrhythmogenic right ventricular cardiomyopathy (ARVC)	3	22	4.92	1.68
	map04960 Aldosterone-regulated sodium reabsorption	2	9	8.02	1.62
	map04066 HIF-1 signaling pathway	3	30	3.61	1.33
Q2	map03010 Ribosome	9	55	6.1	5.24
	map00062 Fatty acid elongation	2	6	12.42	2.01
	map01040 Biosynthesis of unsaturated fatty acids	2	7	10.65	1.87
Q3	map05203 Viral carcinogenesis	16	45	2.63	3.96
	map04520 Adherens junction	11	27	3.02	3.44
	map00630 Glyoxylate and dicarboxylate metabolism	4	5	5.92	2.84
	map05130 Pathogenic Escherichia coli infection	17	66	1.91	2.29
	map04510 Focal adhesion	12	42	2.12	2.05
	map04144 Endocytosis	17	72	1.75	1.73
	map04390 Hippo signaling pathway	7	23	2.25	1.58
	map05210 Colorectal cancer	5	14	2.64	1.52
	map04670 Leukocyte transendothelial migration	10	39	1.9	1.5
	map05100 Bacterial invasion of epithelial cells	7	24	2.16	1.48
	map04530 Tight junction	12	50	1.78	1.47
	map00260 Glycine, serine and threonine metabolism	3	6	3.7	1.45
	map04141 Protein processing in endoplasmic reticulum	15	66	1.68	1.41
	map03050 Proteasome	8	29	2.04	1.33
	map04666 Fc gamma R-mediated phagocytosis	8	29	2.04	1.33
	map00061 Fatty acid biosynthesis	2	3	4.94	1.3
Q4	map04744 Phototransduction	4	4	7.7	3.56
	map03010 Ribosome	17	55	2.38	3.54
	map05132 Salmonella infection	7	15	3.59	2.85
	map04145 Phagosome	13	44	2.28	2.6

	map04745 Phototransduction - fly	5	9	4.28	2.55
	map05120 Epithelial cell signaling in Helicobacter pylori infection	8	23	2.68	2.23
	map04015 Rap1 signaling pathway	13	47	2.13	2.22
	map05100 Bacterial invasion of epithelial cells	8	24	2.57	2.1
	map04740 Olfactory transduction	4	8	3.85	1.9
	map05134 Legionellosis	5	12	3.21	1.89
	map04510 Focal adhesion	11	42	2.02	1.77
	map04520 Adherens junction	8	27	2.28	1.77
	map04721 Synaptic vesicle cycle	6	18	2.57	1.67
	map05130 Pathogenic Escherichia coli infection	15	66	1.75	1.65
	map04670 Leukocyte transendothelial migration	10	39	1.97	1.58
	map05110 Vibrio cholerae infection	6	19	2.43	1.56
	map04910 Insulin signaling pathway	7	25	2.16	1.47
	map04922 Glucagon signaling pathway	7	25	2.16	1.47
	map05133 Pertussis	5	15	2.57	1.46
	map04924 Renin secretion	6	20	2.31	1.45
	map04915 Estrogen signaling pathway	8	29	2.12	1.37
	map04142 Lysosome	8	29	2.12	1.37

Table SVI. Protein domain enrichment in each cluster

Cluster	Domain description	Mapping	Background	Fold Enrichment	-log10(Fisher's exact test p value)
**Q2**	PDZ domain (Also known as DHR or GLGF)	2	8	10.41	1.85
	short chain dehydrogenase	2	9	9.25	1.74
**Q3**	UBA/TS-N domain	6	11	4.68	3.2
	14-3-3 protein	4	7	4.9	2.33
	S-100/ICaBP type calcium binding domain	5	11	3.9	2.29
	SH3 domain	4	8	4.29	2.07
	Calponin homology (CH) domain	6	18	2.86	1.89
	Ca2+ insensitive EF hand	3	5	5.14	1.89
	XPC-binding domain	2	2	8.57	1.87
	ThiF family	3	6	4.29	1.62
	Spectrin repeat	4	11	3.12	1.52
	BRO1-like domain	2	3	5.72	1.43
	CD80-like C2-set immunoglobulin domain	2	3	5.72	1.43
	Cullin protein neddylation domain	2	3	5.72	1.43
	ENTH domain	2	3	5.72	1.43
	Lamin Tail Domain	2	3	5.72	1.43
	Tubulin C-terminal domain	3	7	3.67	1.42
	Tubulin/FtsZ family, GTPase domain	3	7	3.67	1.42
**Q4**	VHS domain	4	8	4.19	2.03
	GAT domain	3	5	5.03	1.86
	XPC-binding domain	2	2	8.39	1.85
	UBA/TS-N domain	4	11	3.05	1.49
	Thyroglobulin type-1 repeat	2	3	5.59	1.41
	Tubulin C-terminal domain	3	7	3.6	1.39
	Tubulin/FtsZ family, GTPase domain	3	7	3.6	1.39
	Ubiquitin family	4	12	2.8	1.35

Table SVII. Interaction network

Term ID	Term Description	Observed Gene Count	Background Gene Count	Strength
CL:14966	GTP hydrolysis and joining of the 60S ribosomal subunit, and Protein export	29	153	1.83
CL:14967	GTP hydrolysis and joining of the 60S ribosomal subunit, and Protein export	28	148	1.83
CL:14978	Peptide chain elongation	23	84	1.99
CL:14980	Peptide chain elongation	22	78	2
CL:14982	Peptide chain elongation	21	73	2.01
CL:14985	Viral mRNA Translation	19	58	2.07
CL:15205	eukaryotic translation initiation factor 3 complex, and eukaryotic translation initiation factor 2 complex	5	18	1.99
CL:13761	Proteasome	4	37	1.58
CL:15206	eukaryotic translation initiation factor 3 complex	3	11	1.99
CL:13763	Proteasome	3	21	1.71
CL:15207	eukaryotic translation initiation factor 3 complex, eIF3m, and RNA polymerase I-associated factor PAF67	2	6	2.07
CL:15227	mixed, incl. eukaryotic translation initiation factor 2 complex, and 40S ribosomal protein SA	2	7	2.01
CL:1604	RHO GTPases Activate WASPs and WAVEs, and VASP tetramerisation	3	41	1.42
CL:13765	Proteasome alpha-type subunit, and proteasome regulatory particle	2	10	1.85
CL:15108	Diamond-Blackfan anemia, and 60s Acidic ribosomal protein	2	10	1.85
CL:1606	RHO GTPases Activate WASPs and WAVEs	2	19	1.57
CL:19155	clathrin coat of coated pit, and ANTH domain	2	20	1.55
CL:989	mixed, incl. ECM-receptor interaction, and cell-substrate junction assembly	3	86	1.09
CL:19104	mixed, incl. Clathrin-mediated endocytosis, and neurotransmitter secretion	4	200	0.85
CL:19106	Clathrin-mediated endocytosis, and AP-type membrane coat adaptor complex	3	112	0.98
CL:1083	mixed, incl. Laminin interactions, and p21 activated kinase binding domain	2	39	1.26
